# Post-operative pain and patient satisfaction after multimodal analgesia: Questionnaire-based chart review

**DOI:** 10.6026/973206300221954

**Published:** 2026-04-30

**Authors:** Leander Pradeep James, Sanathraj Patlu Devaraj, Harsh V Salankar

**Affiliations:** 1 Department of Hepato-Pancreato-Biliary surgery, Christian Medical College, Vellore, Tamil Nadu, India; 2 Department of Medicine, Bangalore hospital, Kengeri, Karnataka, India; 3 Department of Pharmacology, N.K.P. Salve Institute of Medical Sciences & Research Centre and Lata Mangeshkar Hospital, Nagpur, India

**Keywords:** Post-operative pain, multimodal analgesia (MMA), pain management, patient satisfaction, perioperative care, chart review, tertiary hospital study

## Abstract

Post-operative pain remains inadequately controlled in many tertiary hospitals despite widespread adoption of multimodal analgesia protocols and real-world adherence often differs from guideline recommendations. Therefore, it is of interest to evaluate post-operative pain intensity, multimodal analgesic use and patient satisfaction among 220 adults undergoing elective surgery. Mean VAS scores peaked at 6 hours (6.3 ± 1.4) and declined significantly by 24 hours (3.1 ± 0.9) and 48 hours (1.7 ± 0.6), with triple-drug combinations producing superior pain reduction compared to dual or single-agent regimens (p < 0.05). Higher satisfaction was strongly correlated with lower pain scores (r = -0.67, p < 0.001) and delayed analgesic administration was significantly associated with breakthrough pain episodes. Thus, we show that protocol adherence and timely multimodal administration, rather than regimen presence alone, determine post-operative comfort and functional recovery.

## Background:

Post-surgical pain continues to be the most frequent negative experience after surgery and remains poorly controlled, despite clinical guidelines [1]. Poorly controlled post-operative pain may lead to increased sympathetic activation, delayed mobilization, pulmonary complications and longer length of stay in hospital and lower levels of satisfaction for patients [1, 2]. Current practice in the perioperative setting encourages the use of multimodal analgesia to provide improved effectiveness through a combination of agents that target all nociceptive pathways while minimizing the incidence of adverse effects associated with opioids [3, 4]. Typical multimodal approaches utilize a combination of NSAIDs, acetaminophen, regional techniques and limited opioids, which have all been shown to perform better compared to monoclonal therapy in clinical trials [5, 6]. Nevertheless, the implementation of these protocols into practice has not been consistent. The efficacy of implementation into clinical practice depends on timely delivery of medications, adherence to standard algorithms, documentation quality and the manner in which patients are assessed for pain [7, 8]. In addition, in the context of tertiary care settings, barriers to achieving successful multimodal implementation of analgesic regimens include delays in medication delivery and incomplete adoption of multimodal techniques. As patient satisfaction has become an important quality measure in perioperative practice, there is evidence that patient satisfaction is much more highly correlated with the responsiveness and effectiveness of a patient's pain management than with the actual pain scores reported [9, 10]. Therefore, it is of interest to evaluate the post-operative pain response patterns, multimodal analgesic compliance and patient level of satisfaction will be performed using integrated questionnaire and chart review methods in the tertiary hospital.

## Materials and Methods:

The study was done in a teaching hospital for surgery (a large hospital that teaches how to do surgery) over a year. Of 220 adult patients between 18-70 years of age who underwent an elective general, gastrointestinal, or orthopaedic procedure with either regional or general anaesthesia, all were eligible for inclusion in the study by consecutive enrolment. The researchers eliminated from the study patients with chronic pain syndromes; patients who had been diagnosed with a psychiatric illness, or were using opioids or sedatives prior to their procedure were excluded to avoid any opportunities for confounding factors to affect study outcomes. The Institutional Ethics Committee approved of the study protocol and all participants provided their written informed consent. There were two primary elements of data collection used in this study: 1) an initial, structured patient-reported questionnaire that asked patients to rate the severity of their pain using the visual analogue scale, on a scale from 0 (no pain) to 10 (the worst pain imaginable); and 2) the patient's surgical record as it pertains to treatment, pain management, history, etc., The patients' pain level was measured at six hours post-surgery, twelve hours, twenty-four hours and forty-eight hours post-surgery; at rest and when moving; and at each measurement time point the patients were asked about their level of satisfaction (using a 5-point Likert Scale) with the amount of pain they experienced overall. The other data source was the patients' surgical chart, which provided information about the types of medications, the timing of administration, the frequency of administration and what types of combinations were administered within the first forty-eight hours after surgery. All medications used in this study were classified as Multimodal Analgesia (two or more from different drug classifications used together). All multi-drug combinations used to treat the patients involved a combination of any of the following: non-steroidal anti-inflammatory drugs (NSAIDs), acetaminophen, opioids and local anaesthetics or adjunctive agents. The extent to which the hospitals' prescribed treatment protocols complied with institutional standards was evaluated by comparing the regimens patients were prescribed and the times the medications were administered to the hospital institutional guidelines. Demographic and clinical characteristics were summarised by descriptive statistics. Independent t-tests (for continuous data) and Chi-square tests (for categorical data) were conducted to assess statistical differences between regimen categories. Pearson's correlation coefficient was calculated to assess the statistical relationship between patient pain scores and the level of patient satisfaction. The level of statistical significance was set at p<0.05 and all data were analysed using SPSS version 26.0.

## Results:

Data were collected on 220 patients that were undergoing elective surgery. The average age of patients was 45.8 ± 13.4 years and 57.3% were male. The majority of surgical procedures performed were gastrointestinal (46.4%), with orthopedic surgeries following (31.8%) and general surgeries last (21.8%). The mean length of time that patients were operated on was 138 ± 46 minutes. Most patients had ASA I or II physical classifications. Post-operative pain peaked at six hours after surgery (mean VAS 6.3 ± 1.4) and then decreased steadily to 3.1 ± 0.9 at 24 hours and 1.7 ± 0.6 at 48 hours after surgery. The most often utilized multimodal regimen consisted of a three-drug combination (68.2%) and resulted in lower VAS pain score than dual or single agent regimens (p < 0.05). The average overall protocol compliance rate for this study was 81.8% of patients enrolled were either satisfied or very satisfied with their pain control. Patients who had delayed analgesic administration also had significantly higher breakthrough pain episodes (p < 0.05) and patients who had lower VAS scores had significantly higher satisfaction scores (r = -0.67, p < 0.001). Table 1 (see PDF) shows that the stated common characteristics among these patients as compared to the national averages are: Age (31-50 years) = 47.3%; Sex=57.3% male; Type of surgical procedure=46.4% underwent gastrointestinal surgery; ASA classification of I and II (89%) indicates that these patients had lower risks for elective surgery. Table 2 (see PDF) shows that among those receiving drugs as part of multimodal therapy, the proportion of patients receiving a triple drug regimen was 68.2%; dual therapy 21.8%; and monotherapy (only one drug), 5.5%. This reflects a high degree of combination versus monotherapy in analgesic management. In Table 3 (see PDF), we see a steady decline in mean VAS scores from 6.3 ± 1.4 at hour 6 to 1.7 ± 0.6 at hour 48 indicating a significant reduction over time in post-operative pain as reported by patients. From Table 4 (see PDF) we see that 81.8% of cases were completely compliant with institutional multimodal protocols while the remaining 18.1% were partially or non-compliant. Table 5 (see PDF) shows that 70.9% of patients were either satisfied or very satisfied with their post-operative pain control and only 10.9% reported dissatisfaction. In Table 6 (see PDF) we see a graded inverse correlation between patient pain intensity and patient satisfaction. Here we see that the mean VAS for the satisfied patient was 1.8 while the mean VAS for the dissatisfy patient was 6.1, which is statistically significant (p < 0.001). From Table 7 (see PDF) we see that the incidence of breakthrough pain events increased from 14% when analgesics were given on schedule to 38% when there was a delay in administering analgesia of more than 30 minutes, which indicates the importance of timeliness in pain management. In Table 8 (see PDF) we see a statistically significant difference (p < 0.05) between the drugs in triple drug regimens and the drugs in dual or single drug regimens at both 24 and 48 hours with regard to mean VAS scores, confirming that the triple drug regimen provided superior analgesic efficacy. In Table 9 (see PDF), we see that although there were adverse effects observed, they were generally mild. Nausea occurred in 9.1% of patients and 76.3% of patients did not report any adverse effects. Table 10 (see PDF) shows that patients receiving multimodal medication were able to mobilize (get out of bed) earlier (11.6 ± 3.2 hours post-operatively) than patients receiving single agent medication (18.4 ± 4.6 hours post-operatively), indicating that patients receiving multimodal therapy experienced an improved recovery with respect to functional status.

## Discussion:

Findings of this investigation convey the significant benefit by using high adherence to protocol guidelines and addressing medication quickly after surgery for post-operative pain management, patient satisfaction and earlier mobility and function after surgery through the use of a multimodal analgesia approach. Analgesic pain levels were highest in the first six hours and then decreased steadily within 48 hours which correlates with the anticipated inflammatory and nociceptive response after elective surgery, supporting the use of multimodal analgesia for pain management. The findings show that using three drugs resulted in lower mean VAS scores compared to two-drug therapy or one-drug therapy, indicating that the principle of targeting multiple nociceptive pathways creates the maximum therapeutic effect from using a combination of medications [11, 12]. Furthermore, support for using multimodal non-opioid medications as a standard of care in the perioperative environment is reaffirmed through current literature [13, 14]. A strong inverse correlation between pain intensity and satisfaction levels (r = -0.67) validates that the adequacy of pain control is a primary factor in determining the perceived quality-of-care for patients. Recent studies have indicated that patients' perception of satisfaction regarding pain management is primarily based upon both the consistency and responsiveness of the pain management provided, rather than only based upon pain severity alone [15, 16]. Patients in this study stated that their dissatisfaction with pain control was primarily due to delayed administration of analgesics and breakthrough pain, indicating the importance of both the method of medication selection and also the timing of when they receive their medication. This demonstrates the need for protocol compliance to provide for adequate pain management to all patients unless it is implemented effectively and operationally [17]. Although the majority of patients were compliant with protocol guidelines (81.8%), almost one- fifth of the patients studied did not comply with protocol guidelines completely or partially. In this group, patients that did not comply were at an increased risk for experiencing breakthrough pain. This is consistent with research in the area of implementation studies, which has shown that small lapses in the timing of receiving prescribed medications have a substantial impact on early recovery metrics [17, 18]. This study evaluates the impact of real-life delivery on clinical and experiential outcomes via a combination of chart-based documentation and patient reported outcomes (PROs). Although earlier studies primarily focused on the effects of individual analgesic regimens, this analysis evaluated the combined effects of the following factors within the same framework: 1. Adherence, 2. Timing, 3. Pain trajectory, 4. Satisfaction. This method clarifies the operational factors that influence multimodal success. In patients receiving multimodal therapy, the time to ambulation was significantly earlier than in those receiving single-agent therapy. This finding supports the principles of Enhanced recovery after surgery (ERAS), which advocate the use of effective analgesia as a means of promoting ambulation, decreasing the risk of pulmonary complications and decreasing length of hospital stay [19, 20]. The difference in time to mobilization of almost seven hours between multimodal and single agent therapy represents a clinically relevant difference, as well as an indication that multimodal analgesic regimens may improve functional recovery in conjunction with providing symptomatic relief. The side effects associated with combination regimens were mostly mild and infrequent, supporting the continued use of combination regimens when appropriately dosed and underscoring the importance of the current guidelines that promote opioid-sparing strategies [3, 4]. The absence of serious adverse events further strengthens the risk/benefit ratio associated with the structured use of multimodal protocols in elective surgical populations. This study provides additional evidence that bridges the gap between controlled trial outcomes (efficacy) and actual outcomes (effectiveness). Controlled studies tend to demonstrate optimal results under highly monitored conditions, while routine clinical settings may have more documentation errors as well as communication errors. As seen in this analysis, there is a direct relationship between the timing of medication administration and the frequency of breakthrough pain, providing objective evidence that the operational efficiency of a clinic has an impact on the therapeutic benefit of the analgesic being utilized. Additionally, this study extends the body of literature regarding the impact that adherence to medication protocols and the timing of medication administration have as independent contributors to patient satisfaction and patient recovery, in addition to the pharmacologic agent that was selected. The limitations of this study include the fact that it is a single center study and did not evaluate long-term outcomes. In addition, although a pain assessment tool was used to standardize the measurement of pain, it remains a subjective perception of each patient. Future studies that include multiple centers and utilize digital analgesic tracking and real-time alert systems will better optimize the precision of medication delivery. Through combined use of systematic multimodal analgesia, strict timing adherence and effective communication, multimodal analgesia can greatly improve post-operative pain control and support patient-centered recovery in tertiary surgical practice.

## Conclusion:

Systematic multimodal analgesia significantly reduces post-operative pain, improves patient satisfaction and facilitates earlier mobilization when administered in a timely and protocol-adherent manner. Delays in analgesic delivery are independently associated with breakthrough pain and reduced satisfaction despite appropriate regimen selection. Strengthening protocol adherence, documentation accuracy and perioperative communication is essential to optimize real-world effectiveness of multimodal pain management in tertiary surgical practice.
